# Prolonged lipopolysaccharide‐induced illness elevates glucagon‐like peptide‐1 and suppresses peptide YY: A human‐randomized cross‐over trial

**DOI:** 10.14814/phy2.15462

**Published:** 2022-09-18

**Authors:** Katrine Brodersen, Maike Mose, Ulla Ramer Mikkelsen, Jens Otto Lunde Jørgensen, Michael Festersen Nielsen, Niels Møller, Anne‐Marie Wegeberg, Christina Brock, Bolette Hartmann, Jens Juul Holst, Nikolaj Rittig

**Affiliations:** ^1^ Department of Surgery Viborg Regional Hospital Viborg Denmark; ^2^ Medical/Steno Aarhus Research Laboratory Aarhus University Hospital, Aarhus University Aarhus Denmark; ^3^ Steno Diabetes Center Aarhus Aarhus University Hospital Aarhus Denmark; ^4^ Arla Foods Ingredients Group P/S Viby Denmark; ^5^ Department of Endocrinology and Internal Medicine Aarhus University Hospital Aarhus Denmark; ^6^ Mech‐Sense, Department of Gastroenterology and Hepatology Aalborg University Hospital Aalborg Denmark; ^7^ Steno Diabetes Center North Denmark Aalborg University Hospital Aalborg Denmark; ^8^ Department of Biomedical Sciences and Novo Nordisk Foundation Center for Basic Metabolic Research University of Copenhagen København Denmark

**Keywords:** endotoxemia, gastrointestinal hormones, gastrointestinal motility, gastrointestinal transit times, inflammation, wireless motility capsule

## Abstract

Severe systemic inflammation is associated with nausea, loss of appetite, and delayed gastric emptying, which increases hospitalization admission length and mortality rate. There is a lack of human controlled studies exploring gastric emptying rates and underlying mechanisms during inflammatory conditions. We aimed to investigate if systemic inflammation in young men delays gastro‐intestinal transit times, lowers motility, and affects gastrointestinal hormone secretion. This substudy of a randomized crossover trial investigated eight healthy young men on two separate occasions; (I) following an overnight fast (healthy conditions/HC) and (II) fasting and bedrest combined with two lipopolysaccharide (LPS) injections of 1 ng kg^−1^ following an overnight fast and 0.5 ng kg^−1^ following another 24 h (systemic inflammation/SI). A standardized protein beverage and a SmartPill capsule (a wireless gastrointestinal monitoring system) were swallowed during each occasion. Whole gut transit time was comparable between HC and SI. SI decreased gastric mean pressure peak amplitude (*p* = 0.04) and increased pH rise across the pylorus and small bowel pH (*p* = 0.02) compared with HC. Glucagon‐like peptide‐1 was elevated during SI compared with HC (*p* = 0.04). Peptide YY was lower during SI compared with HC (*p* = 0.007). Prolonged LPS exposure combined with fasting and bedrest elevated glucagon‐like peptide 1 concentrations, which may play a role for the nausea and loss of appetite typically associated with SI.

## Graphical abstract

Inflammatory conditions prolong gastrointestinal transit times, but randomized humane studies are lacking. During standardized inflammation motility was decreased, glucagon‐like peptide‐1 was elevated and peptide YY lowered. In conclusion, gastrointestinal hormones may take part in the nausea and malnutrition associated with prolonged systemic inflammation.

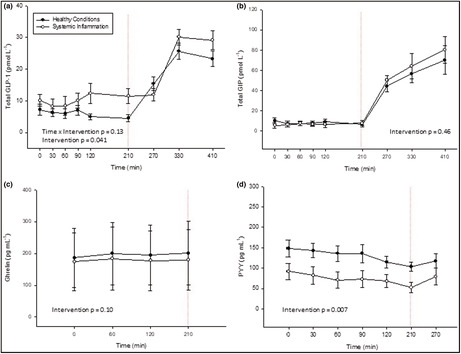

## 1 INTRODUCTION

Microbial infections causing systemic inflammation (SI) are often associated with nausea and loss of appetite. Around two‐thirds of critically ill patients with sepsis have delayed gastric emptying time (GET) and the prevalence increases with disease severity (Nguyen et al., 2007). Delayed GET is associated with elevated risk of aspiration, increased need of intensive care, prolonged hospital admission, and elevated mortality rate (Nguyen et al., 2007; Van Leeuwen et al., 1994). Inflammation that is more moderate leads to nausea, reduced food intake, immobilization as well as negative energy balance with elevated muscle wasting, which is subsequently associated with reduced quality of life and increased mortality in elderly and/or chronically ill patients (Liebau et al., 2015; Welch et al., 2018). However, these important clinical results are often obtained in a heterogeneous patient population and are poorly controlled (e.g., with respect to time of inflammation onset and disease severity). Gastrointestinal motility during conditions with SI has mainly been explored in animal studies, showing a disruption in normal motor functions and a delay in gastric emptying and colonic transit time (Calatayud et al., 2001; Cullen et al., 1995; Cullen et al., 1997). Human studies are sparse and have primarily evaluated gastrointestinal disorders in outpatient settings or examined GET in postoperative or critically ill patients in the intensive care unit (ICU). These studies have shown delayed GET in line with clinical experience (Nguyen et al., 2007). Lipopolysaccharide (LPS) shed by gram‐negative bacteria activates the innate immune system through toll‐like receptor 4 activation (Zhang et al., 2020). Intravenous bolus infusion of LPS has been used as an experimental approach in humans to mimic the systemic inflammatory response during the initial phase of sepsis and affect many other organs than the immune system through yet unknown mechanisms (van Lier et al., 2018).

The gold standard for measuring gastrointestinal transit time is scintigraphy, either as gastric scintigraphy or as whole gut scintigraphy, the latter lasting several days (Bonapace et al., 2000). Motility is most often assessed by manometry (Cassilly et al., 2008). These techniques are laborious, costly, may involve radiation, and transport to facilities, which pose ethical and logistic challenges in clinical studies. Alternatives for measuring gastrointestinal transit times and motility include ingestion of a capsule, that measures whole gut transit times and motility in an ambulant setting without radiation exposure. This relative new method correlates well with scintigraphy and antroduodenal manometry measurements (Cassilly et al., 2008; Kuo et al., 2007).

The aim of this randomized crossover exploratory study was to investigate the effects of prolonged, well‐controlled moderate SI on gastrointestinal transit time, motility, and hormone secretion in young men.

## 2 MATERIALS AND METHODS

### 2.1 Study approval

The study was approved by the Central Denmark Region Committees on Biomedical Research Ethics (ID: 1‐10‐72‐69‐19), and was registered at the Danish Data Protection Agency and at ClinicalTrials.gov (NCT04064268). The study was conducted in accordance with the Declaration of Helsinki. All participants signed an informed consent, prior to inclusion into the study.

### 2.2 Design and participants

Our data originate from a larger randomized controlled crossover study with 8 male participants who were investigated on three separate occasions to explore the effect on protein metabolism of (i) protein supplementation during healthy conditions (HC), (ii) protein supplementation during systemic inflammatory conditions (SI), and (iii) ketone supplementation during systemic inflammatory conditions (Mose et al., 2021). This study only involves data from the two first intervention arms. The inclusion criteria in the study were male gender, aged between 20 and 40 years and a body mass index (BMI) between 20 and 30 kg/m^2^. The participants were healthy without regular intake of medicine and were nonsmokers. Prior to inclusion, they were screened with a medical interview and physical examination as well as electrocardiography and blood tests: C‐reactive peptide (CRP), leukocytes, hemoglobin, thrombocytes, creatinine, alanine, aminotransferase, bilirubin, alkaline phosphatase, hemoglobin A1c, thyrotropin, and cholesterol.

Exclusion criteria were ongoing signs of infection (e.g., fever or elevated CRP) or gastrointestinal conditions including prior abdominal surgery.

Participants were instructed to abstain from alcohol and strenuous physical activity for 48 h before each visit, and to consume food with 50–60 E% from carbohydrates, 10–20 E% from protein and max 30 E% from fat. They fasted overnight prior to the study days and were transported to the research facilities by a motorized vehicle.

### 2.3 Interventions

Participants were investigated following an overnight fast, on two separate occasions in random order (with 6–8 weeks between visits);

- HC.
- Prolonged LPS‐induced SI
  LPS injection (1 ng kg^−1^, time = −24 h), fast and immobilization (prestudy day).
  LPS injection (0.5 ng kg^−1^, time = 0) (study day).

**FIGURE 1:**
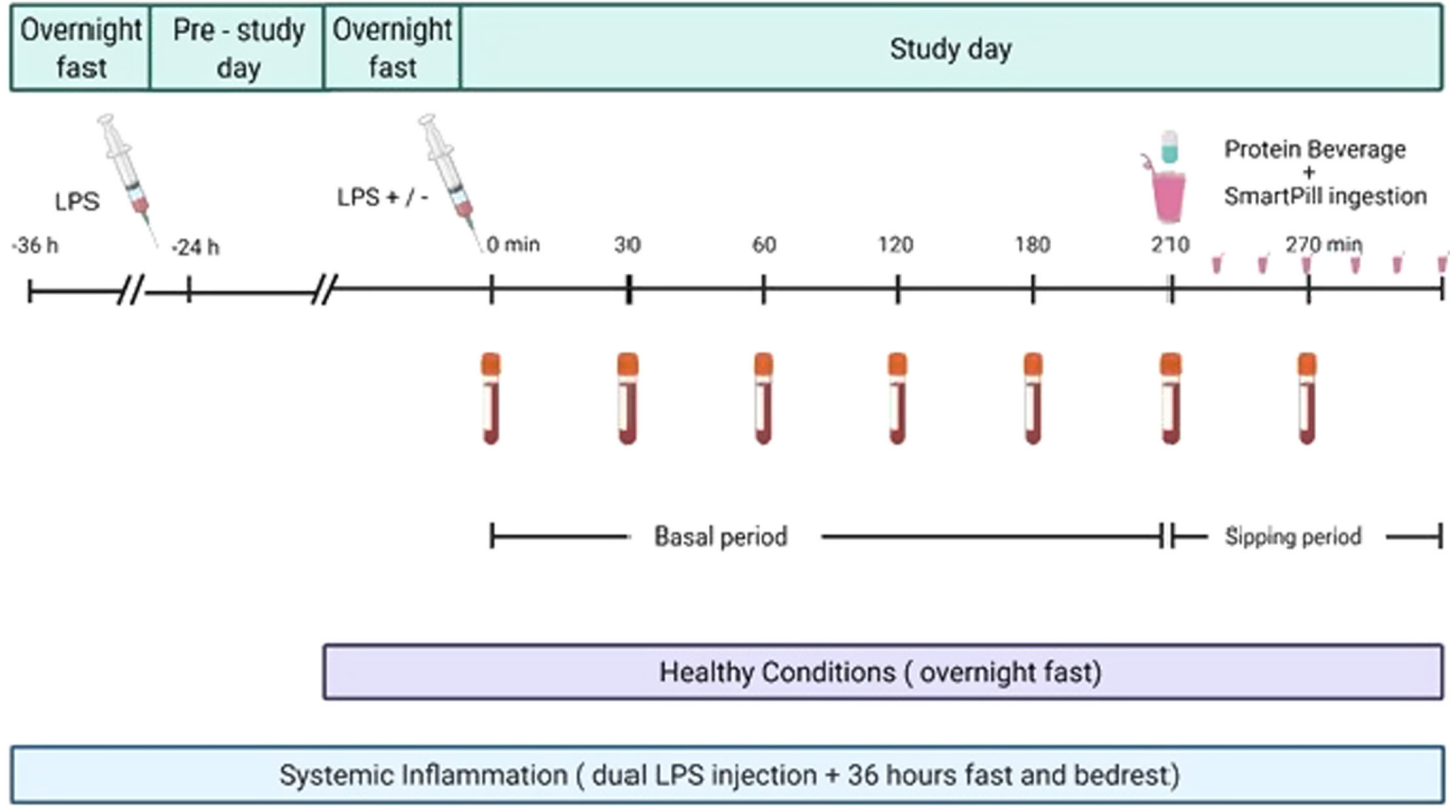
Flowchart of the study days. Flowchart showing healthy conditions and prolonged systemic inflammatory conditions. LPS, lipopolysaccharide. Created with BioRender.

Heart rate and axillary temperature were measured every hour. Blood samples were obtained consecutively during a 7‐h period comprising a 3.5 h fasting period (basal) and a 3.5 h postprandial (sipping) period. A beverage consisting of 15 gram β‐lactoglobulin protein (produced by Arla Foods Ingredients)+6.7 g carbohydrate from maltodextrin +14.1 g lipid from sunflower oil and milk and tap water (total volume 188 ml) was ingested as a bolus (time = 210) followed by fractions of a beverage with the same composition every 20 min (sipping period; time 210–420). In total, 501 kcal was ingested in the form of 45 g β‐lactoglobulin protein, 20 g maltodextrin and 28.2 g lipid from sunflower oil and milk. A SmartPill was swallowed immediately following the bolus. The study flowchart is shown in Figure 1.

Data relating to SI, glucose, insulin, glucagon, and protein metabolism are published elsewhere (Mose et al., 2021).

After 7 h, each participant received a standard sandwich meal and returned home to their normal lifestyle carrying the SmartPill receiver (see below).

### 2.4 SmartPill

**FIGURE 2:**
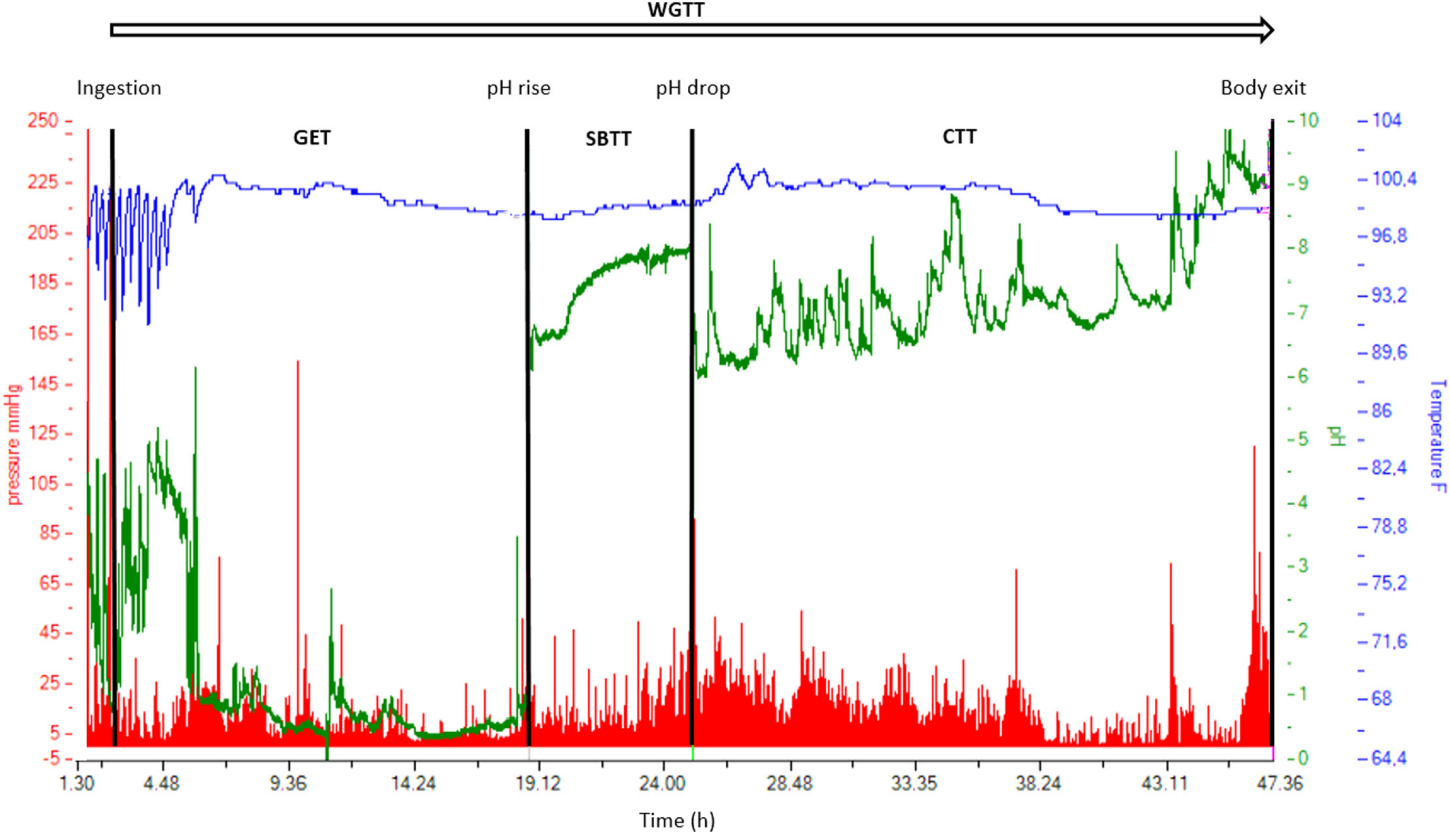
SmartPill output. An example of the SmartPill output measure is shown with pH (green vertical axis), temperature (blue vertical axis) and pressure (red vertical axis) during time (horizontal axis). Whole gastrointestinal transit time (WGTT), gastric emptying time (GET), small bowel transit time (SBTT) and colonic transit time (CTT).

An activated and calibrated SmartPill (Given Imaging) capsule was swallowed with 150 ml of tap water immediately after protein beverage ingestion. The SmartPill is a 27 × 12 mm cylindrical shaped plastic capsule with a weight of 4.5 g. It measures pressure in the range of 0–350 mmHg ± 5 mmHg from 0–99 mmHg and ±10% above 100 mmHg. The pH sensor measures between 1–9 ± 0.5 pH unit and temperatures from 20 to 45 ± 1°C. Via radiofrequency, data are transmitted to a portable receiver on the abdomen of the participant. After SmartPill expulsion, the receiver is docked to a computer and the MotiliGI (SmartPill‐software, version 3.0.20, Given Imaging) aids in the retrieving and analysis of the results. A typical output is shown in Figure 2.

### 2.5 Data analysis

Each SmartPill trace displays pH, temperature, pressure, and time. Two blinded investigators analyzed each trace and any incoherency between estimates was discussed and settled by the blinded study group.

Anatomical landmarks were defined as previously described (Evans et al., 1988; Fallingborg et al., 1998; Sarosiek et al., 2010; Zarate et al., 2010). In brief, the GET was the time interval between ingestion, defined by an abrupt temperature increase to >37° C and the passage through pylorus, defined by a sharp rise in pH by more than two units. Small bowel transit time (SBTT) was the time interval from passage through the pylorus to passage through the ileocecal junction (ICJ), defined by a sustained drop in pH by more than 1 pH unit for at least 10 min. Colonic transit time (CTT) was the time interval from the passage through ICJ to SmartPill expulsion, defined by a sharp drop in temperature (Figure 2). Proper assessment of pH values requires gastric retention of the SmartPill for at least 10 min.

### 2.6 Blood samples

Blood samples were placed on ice, centrifuged, and stored at −20°C or − 80°C as appropriate and analyzed in batches. Leucocytes, neutrophils, CRP, glucagon, tumor necrosis factor α (TNFα), interleukin 6 (IL‐6), and serum insulin were analyzed as previously described (Mose et al., 2021). Acylated ghrelin was analyzed with an enzyme‐linked immunosorbent assay (ELISA) kit A05106, from Bertin Bioreagents. Total PYY was measured using Millipore human total PYY Elisa (cat no EZHPYYT66K; Millipore). Plasma for total glucagon‐like peptide 1 (GLP‐1) and glucose‐dependent insulinotropic peptide (GIP) was extracted in a final concentration of 70% ethanol before analysis. Total GLP‐1 was measured as described in (Orskov et al., 1994) using a radioimmunoassay (antibody code no 89390) specific for the C‐terminal part of the GLP‐1 molecule and reacting equally with intact GLP‐1 and the primary (N‐terminally truncated) metabolite. Total GIP concentration was measured with a radioimmunoassay using an antibody directed toward the C‐terminus (code no. 80867), which reacts fully with intact GIP and N‐terminally truncated forms as described in (Lindgren et al., 2011). Sensitivity for both assays was below 1 pmol L^−1^, and intra assay coefficient of variation below 10%. Where relevant, assays were carried out in accordance with the manufacturer's instructions and all quality controls were within the required limits.

### 2.7 Statistics

Statistical analyses were carried out in STATA 14 (StataCorp LLC) and graphs in SigmaPlot 11 (Systat Software). Data are expressed as mean ± standard deviations or median (range) and a *p* < 0.05 was considered significant. For comparison between interventions a paired *t*‐test was performed. QQ plots and Bland–Altman plots were inspected to control for model assumptions. If model assumptions were violated, logarithmic transformation was applied. Gastrointestinal hormones were examined with a linear repeated measure mixed model with two factors: intervention (SI vs. HC) and time and any interaction between them (intervention × time). Residual plots were inspected for equal variance.

## 3 RESULTS

### 3.1 Participants

The participants had a median age of 24.5 years (21–27) and a median BMI of 23.3 kg/m^2^ (20.8–29.9) as previously reported (Mose et al., 2021). All interventions were well tolerated except for one participant reporting an episode of diarrhea (during SI). No serious adverse events were recorded and all participants completed the study.

### 3.2 LPS‐induced illness

The systemic effect of the initial dose of LPS (1 ng kg^−1^ at time point −24 h) has already been reported elsewhere (Modrzynska et al., 2021), and included a marked increase in heart rate (peak increase ≈ 25 beats per min), temperature (peak increase ≈ 2°C), inflammatory cytokines (peak increase in TNF‐α ≈ 150 pg ml^−1^ and IL‐6 ≈ 400 pg ml^−1^), and symptom scores (Table 1). We have previously validated this human model of SI and showed it causes insulin resistance (Mose et al., 2020). In this study, we added a second dose of LPS to prolong the inflammatory response.

**TABLE 1:** Vital parameters and inflammatory markers

Measures	Unit	HC	SI	Absolute difference	*p*‐value
Vital parameters					
Heart rate	Beats min^−1^	58 (50 to 66)	64 (60 to 87)	6.5 (−3 to 27)	0.045
Axillary temperature	°C	36.1 ± 0.4	36.5 ± 0.3	0.4 ± 0.3	0.03
Inflammatory markers					
Leucocytes	×10^9^ L^−1^	3.8 ± 0.9	9.8 ± 1.7	6.0 ± 6.9	<0.001
C‐reactive peptide	mg L^−1^	1 (0.1 to 3)	22 (19 to 48)	22 (18 to 46)	<0.001
Interleukine‐6	pg ml^−1^	5.7 (0.3 to 17.1)	16.8 (1.6 to 96.8)	11.1 (1.3 to 86.5)	<0.001
Tumor necrosis factor‐α	pg ml^−1^	6.5 ± 5.8	17.1 ± 10.4	10.6 ± 5.7	0.001

*Note*: Vital parameters and inflammatory markers during healthy conditions (HC) and during systemic inflammation (SI, dual lipopolysaccharide exposure combined with a 36 h fast and bedrest). Mean ± standard deviation or median (min–max), absolute difference and the *p*‐value from a paired *t*‐test.

Following the second dose of LPS (0.5 ng kg^−1^ at time point 0 h), there was a more moderate response with a mean increase in heart rate of 6 beats per min (*p* = 0.045), a 0.4°C increase in body temperature (*p* = 0.03), a 6 × 10^9^ L^−1^ increase in plasma leucocyte count (*p* < 0.001), a 22 mg L^−1^ increase in CRP (*p* < 0.001), an 11 pg mL^−1^ increase in IL‐6 levels (*p* < 0.001), and a 10.6 pg mL^−1^increase in TNF‐α concentrations (*p* = 0.002). Furthermore, mean insulin levels increased by 25 pmol L^−1^ (*p* = 0.02) and mean glucagon levels by 6 pmol L^−1^ (*p* = 0.0001; Mose et al., 2021).

### 3.3 Transit times, motility, and pH data

The WGTT tended to be 7.3 ± 12.2 h longer during SI compared with HC (*p* = 0.13; Table 2).

**TABLE 2:** Transit times, motility, and pH data

Measures	Unit	HC	SI	Absolute difference	% Difference	*p‐*value
Transit times						
Gastric emptying time	min	558 ± 485	689 ± 530	131 ± 557	23.5	0.53
Small bowell transit time	min	323 ± 88	290 ± 96	‐34 ± 70	−10.4	0.22
Colon transit time	min	799 ± 629	1141 ± 652	342 ± 850	42.8	0.29
Whole gut transit time	min	1681 ± 808	2120 ± 709	440 ± 730	26.2	0.13
Motility/contractility						
Gastric motility index	mm Hg s min^−1^	56 ± 41	38 ± 26	−18 ± 43	−31.4	0.29
Small bowell motility index	mm Hg s min^−1^	144 ± 56	131 ± 78	−14 ± 62	−9.6	0.55
Colon motility index	mm Hg s min^−1^	244 (75 to 395)	134 (84 to 640)	−110 (−274 to 245)	−45.1	0.41
Gastric contraction mean peak amplitude	mm Hg	17 (15 to 33)	16 (13 to 21)	−1 (−12 to 1)	−5.9	0.04
Small bowell contraction mean peak amplitude	mm Hg	17 ± 1	18 ± 3	1 ± 3	5.9	0.33
Colon contraction mean peak amplitude	mm Hg	22 ± 4	20 ± 4	−2 ± 6	−7.8	0.43
Gastric contractions	Contractions min^−1^	2.0 ± 1.6	1.5 ± 1.0	−0.5 ± 1.6	−23.9	0.42
Small bowell contractions	Contractions min^−1^	4.2 ± 1.3	3.5 ± 1.9	−0.7 ± 1.7	−17.4	0.26
Colon contractions	Contractions min^−1^	2.2 (1.3 to 5.9)	2.0 (1.3 to 4.8)	−0.2 (−1.1 ‐1.3)	−9.1	0.40
Gastric pressure maximum	mm Hg	292 ± 105	252 ± 138	−40 ± 165	−13.8	0.51
Small bowell pressure maximum	mm Hg	142 (65 to 266)	103 (61 to 245)	−39 (−166 to 96)	−27.5	0.13
Colon pressure maximum	mm Hg	145 ± 39	150 ± 53	5 ± 80	3.4	0.87
pH						
Gastric pH min	pH unit	0.65 ± 0.3	0.72 ± 0.3	0.1 ± 0.5	10.8	0.71
Gastric pH median	pH unit	2.28 ± 1.0	2.39 ± 1.3	0.1 ± 1.4	4.8	0.82
Antroduodenal rise	pH unit	5.78 ± 0.4	6.33 ± 0.3	0.6 ± 0.5	9.7	0.02
Small bowell pH med	pH unit	7.15 ± 0.3	7.44 ± 0.3	0.3 ± 0.3	3.9	0.04
Small bowell pH max	pH unit	7.79 ± 0.3	8 ± 0.4	0.2 ± 0.5	2.7	0.23
Ileocecal junction pH drop	pH unit	1.48 ± 0.5	1.76 ± 0.7	0.3 ± 1.0	18.9	0.45
Colon pH min	pH unit	5.6 ± 0.5	5.6 ± 0.4	0.0 ± 0.5	0.0	1.00
Colon pH med	pH unit	6.94 ± 0.7	7.06 ± 0.7	0.1 ± 1.0	1.7	0.75
Colon pH max	pH unit	8.09 ± 0.5	8.61 ± 0.8	0.5 ± 0.7	5.7	0.09

*Note*: Transit times, contractility measures and pH estimates derived from an ingested SmartPill. The SmartPill was ingested following consumption of a protein beverage bolus followed by a sip regime of the beverage throughout the study. Interventions were healthy conditions (HC) and systemic inflammation (SI, dual lipopolysaccharide exposure combined with a 36 h fast and bedrest). Data are displayed as means ± standard deviation, or medians (min–max) absolute and percentage difference and the associated *p*‐value from a paired *t*‐test.

The gastric mean peak contraction amplitude was 6.0% lower during SI compared with HC (*p* = 0.04).

The median gastric pH was comparable between conditions, but the mean pH across the antroduodenum was 9.7% (*p* = 0.02) higher and the median small bowel pH was 3.9% (*p* = 0.04) higher during SI compared with HC.

### 3.4 Gastrointestinal hormones

**FIGURE 3:**
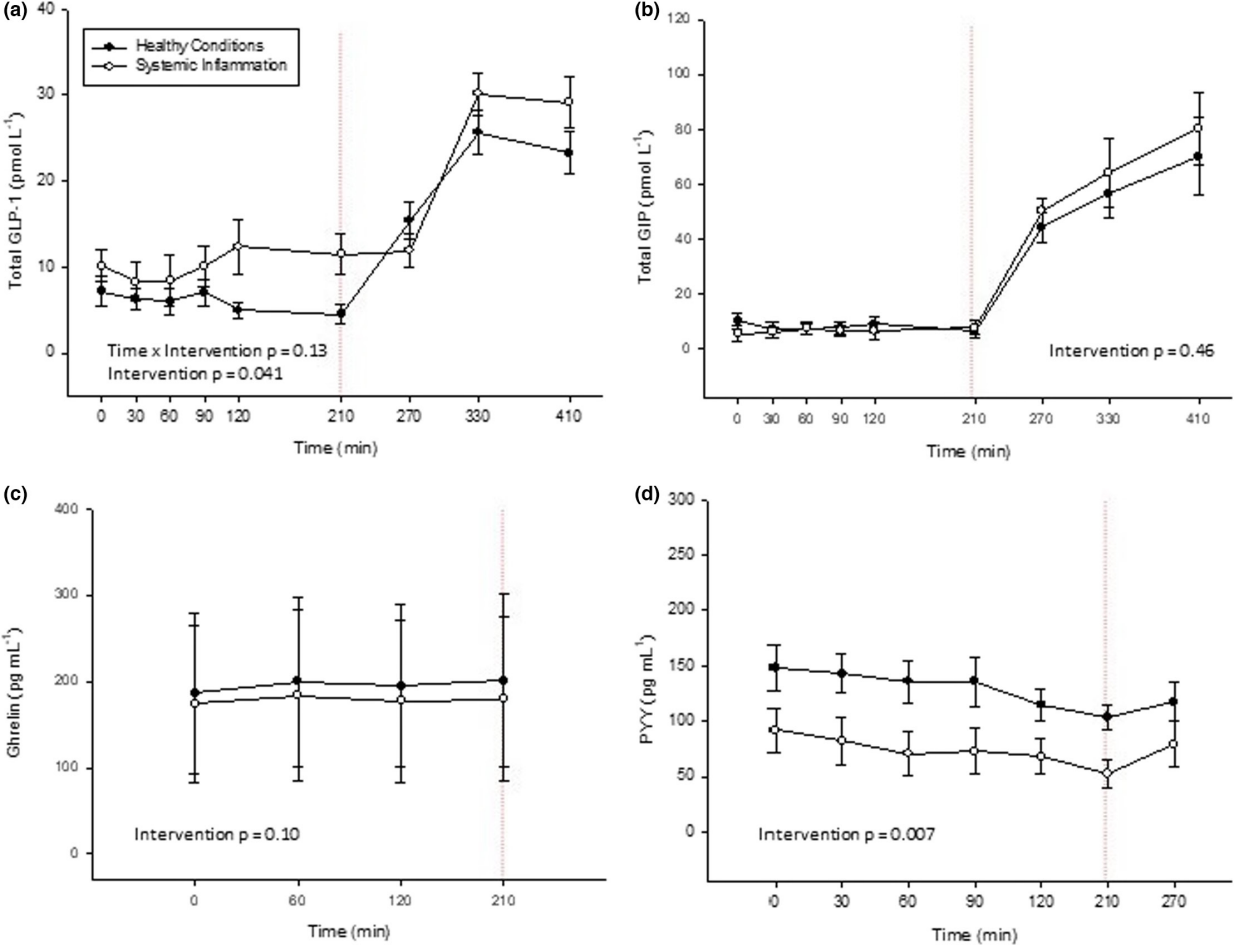
Gastrointestinal hormone. (a) Glucagon‐like peptide 1 (GLP‐1), (b) glucose‐dependent insulinotropic peptide (GIP), (c) Ghrelin and (d) PYY measured during a basal period followed by consumption of a protein beverage bolus followed by a sip regime of the beverage throughout the study. Interventions were healthy conditions (a 12 h overnight fast) and systemic inflammation (dual lipopolysaccharide exposure combined with a 36 h fast and bedrest).

Plasma concentrations of GLP‐1 were higher during SI (intervention, *p* = 0.04) and rose as expected following protein ingestion, with no statistically significant time × intervention interaction (Figure 3a **).**

PYY levels were lower during SI compared with HC throughout the entire trial period (intervention, *p* = 0.007; Figure 3d **)**. Plasma concentrations of acyl ghrelin and GIP were comparable between SI and HC **(**Figure 3b,c **)**.

## 4 DISCUSSION

In this exploratory study, we show that 36 h of SI, fasting, and bedrest elevate plasma GLP‐1 and lower PYY concentrations compared with HC in young men. The systemic inflammatory condition was associated with a decrease in gut motility and regional pH elevations, but did not significantly alter intestinal transit times. However, the individual differences were quite large with a mean delay in whole gut transit times by 7 h ranging from −13 to 24 h, but the study had a limited number of participants. Regardless, the delay of 7 h in whole gut transit time is in line with currently available reports from critically ill humans and animal models of inflammation (Collares, 1997; Cullen, Titler, et al., 1999; Nguyen et al., 2007).

Systemic inflammation activates immune cells in the intestinal wall and afferent neurons resulting in intestinal motility disturbances in animals (Brookes, 1993; De Winter et al., 2009). Activated mast cells, macrophages, and glia cells produce pro‐inflammatory mediators such as IL‐6 and the neurotransmitter nitric oxide (NO), which have been shown to induce delayed GET (Takakura et al., 1997). TNF‐α and NO inhibitors have been shown to reverse the LPS‐induced delay in intestinal transit times in mice (Inada et al., 2006), which indicates that pro‐inflammatory cytokines per se may affect gastrointestinal transit times and motility. Moreover, reduced blood flow during sepsis (Murphey & Traber, 2000) may amplify the pro‐inflammatory response and sympathetic (i.e., dampened parasympathetic) neural activity, causing reduced intestinal motility.

Plasma GLP‐1 concentrations were higher during SI fasting conditions, which underscores the concepts regarding the existence of a basal, meal‐independent GLP‐1 secretion (Holst et al., 2017). An IL‐6 dependent GLP‐1 secretion has been demonstrated in mice but could not be reproduced in humans (Ellingsgaard et al., 2011; Kahles et al., 2014; Lang Lehrskov et al., 2018). TNF‐α was also elevated in our study, but TNF‐α infusion did not alter GLP‐1 secretion in response to an oral glucose tolerance test (Nielsen et al., 2013). It is possible that the L‐cells may be directly activated via their expression of Toll‐like receptors, which may at least partly contribute to the elevated GLP‐1 levels found in our trial (Lebrun et al., 2017).

Previous studies have demonstrated elevated GLP‐1 levels during inflammation (Brakenridge et al., 2019; Perl et al., 2018), but a recent clinical study from our group found that GLP‐1 concentrations decrease during the first 6 h of LPS exposure in healthy young men (Modrzynska et al., 2021). In addition, clinical data from patients with endocarditis, urinary tract infection, noninfectious and postoperative inflammation show divergent correlations between inflammation (using CRP) and GLP‐1. The only subgroup that showed a positive correlation between GLP‐1 and CRP were patients with urinary tract infection with *Escherichia coli*. We used LPS from *E. coli* to induce prolonged SI in this current study and suggest that bacteria strain and duration (acute vs. prolonged) of the inflammation may affect GLP‐1 concentrations.

Studies have shown that GLP‐1 delays GET, decreases hunger sensations, and reduces food intake (Nguyen et al., 2014; Shirazi et al., 2013; Wettergren et al., 1993). Previous studies have demonstrated reduced antroduodenal motility and enhanced pyloric contractions during GLP‐1 infusion in humans (Hellström et al., 2008; Schirra et al., 2000). In line with this, we found a decreased mean peak amplitude of gastric contractions. Thus, our data would be compatible with a theory that GLP‐1 contributes to the nausea and reduced food intake typically accompanying conditions with prolonged SI.

Interestingly, we observed an increase in prandial plasma GLP‐1 during both SI and HC. The increase in GLP‐1 secretion during SI was associated with a ≈1.4 mM increment in prandial plasma glucose concentrations and an increase of ≈100 pmol L^−1^ in plasma insulin compared with no change in prandial plasma glucose and an increase of only ≈40 pmol L^−1^ in plasma insulin during HC conditions (in line with being more insulin resistant during SI), as previously reported (Mose et al., 2020; Mose et al., 2021). Insulin injection in mice induced accelerated jejunal transport independently of hypoglycemia, by activating cholinergic and adrenergic systems (Peddyreddy et al., 2006). We observed a 10% acceleration in jejunal transport during SI, however nonsignificant (*p* = 0.22). It has been shown that LPS infusion accelerates the migrating motor complex propulsion along the jejunum and can be accompanied by a decreased absorption of water, glucose and amino acids in a dose‐dependent fashion in animal studies (Cullen, Doty, et al., 1999; Hellström et al., 1997; Sodeyama et al., 1993). Thus, SI may delay intestinal motility and whole gut transit times but at the same time accelerate SBTT. This may very well induce nausea and reduce food intake, while compromising nutrient and possibly pharmaceutical uptake in the small bowel. These changes in the gastrointestinal environment and movement may also play a role for the catabolic state associated with SI (Mose et al., 2021; Zagli et al., 2008).

Gastric acid secretion is inhibited by GLP‐1, which is in line with our findings of significantly higher median small bowel pH and a larger pH rise from the stomach to the small bowel during SI (Wettergren et al., 1993). Bacterial overgrowth of the upper gastro‐intestinal tract plays a pivotal role in development of inflammatory complications and multiple organ failure in the ICU (Hassoun et al., 2001). Animal studies have shown a decrease in gastric acid secretion during endotoxemia (Cullen et al., 1995), and alkalization of gastric secretion has been linked to bacterial overgrowth in the small bowel and colon of humans (Duncan et al., 2009; Husebye et al., 1992, 1995). Alternatively, alkalization of small bowel pH could also be attributed to the aforementioned decrease in water uptake from the small bowel during SI combined with delayed GET. SI influences the gastrointestinal environment and may therefore play a role for the altered composition of the bacterial flora associated with infections and SI (Alhazzani et al., 2018).

PYY is secreted by L‐cells in the distal ileum in response to nutrients approximately 15 min after meal ingestion and thus before nutrients reach the distal ileum (Price & Bloom, 2014). Our meal contained both protein, fat, and carbohydrates still we were, unable to detect a postprandial rise in PYY concentrations in either group, which may indicate either that the nutrient load was insufficient to stimulate PYY release or the timespan between ingestion and measurements was too short. SI in combination with 36 h of fasting decreased PYY levels throughout the entire study period, an observation in line with the difference seen between obese and lean individuals (Batterham et al., 2003). Thus, these results are in contrast to in vitro and animal studies showing elevated PYY levels during inflammatory conditions (Higashiguchi et al., 1994; Zamir et al., 1992). Increased PYY levels are also reported in critically ill patients (Nematy et al., 2006). Since the secretion of PYY is affected by previous food intake (Chandarana et al., 2009), the downregulation of the anorexigenic hormone PYY in our study may reflect that those participants were fasted for 36 h during SI conditions compared with an overnight fast during HC conditions. The lower concentrations of PYY observed during SI in our study may have had opposing effects on the elevated GLP‐1 concentrations, appetite and intestinal transit times.

The study has several limitations. This is a substudy within a larger clinical study, and it is prone to type 2 errors because of the small number of participants as reflected by many borderline significant *p*‐values. In addition, although the SI produced in our study was comparable to an *E. coli* infection, the level of inflammation was perhaps insufficient to elicit significant effects on transit times and motility. In line with this, there has been reports of dose‐ and time‐dependent effects of LPS on transit times in animals (Ceregrzyn et al., 2001; Collares, 1997), which may also have affected our results (Ceregrzyn et al., 2001). The beverage contained all macronutrients but relatively large amounts of protein as the primary aim of the initial study was to investigate the muscle preserving effects of the beverage during healthy and systemic inflammatory conditions. We cannot exclude that a different nutritional composition of the test meal may have affected intestinal transit times and gastrointestinal hormones. Also, we chose a study design that mimics the typical features (inflammation, low calorie intake, and immobilization) of an infection and compared it with HC (overnight fast). The clinically relevant design is not only a strength but also a limitation as it hinders disentangling whether it is caused by prolonged fasting, immobilization, or inflammation.

We chose to include only healthy young men in order to minimize variability and due to the ethical considerations associated with human studies using LPS. Gender and age affect intestinal transit times (Graff et al., 2001), but we have no reason to believe that our intervention (SI) would affect intestinal transit times or gastrointestinal hormones markedly different in women or older individuals.

The merits of our study, however, include the randomized controlled crossover design with a comprehensive model mimicking prolonged inflammatory conditions in humans, and in our eyes, these data add new important insight into the gastrointestinal changes that occur during these conditions.

## 5 CONCLUSION

In conclusion, we used dual LPS exposure to mimic the first 36 h of bacteremia and showed elevated plasma concentrations of GLP‐1 and lowered plasma concentrations of PYY. The changes in gut hormones were associated with decreased motility and increased pH in the small bowel. These preliminary findings provide new insight into the gastrointestinal adaptions that occur during prolonged SI and GLP‐1 but not PYY may play a role in the nausea associated with this condition.

## AUTHOR CONTRIBUTIONS

Nikolaj Rittig, Niels Møller, Maike Mose, Ulla Ramer Mikkelsen, and Jens Otto Lunde Jørgensen designed the research; Katrine Brodersen and Maike Mose conducted the research; Anne‐Marie Wegeberg, Christina Brock, Bolette Hartmann, Jens Juul Holst, and Katrine Brodersen analyzed data; Katrine Brodersen performed the statistical analysis and wrote the manuscript; All authors contributed with improvement to the manuscript; Nikolaj Rittig was responsible for the final content and all authors approved the final manuscript.

## FUNDING INFORMATION

Financial support came from Aarhus University (Denmark), Department of Surgery, Viborg Regional Hospital, Steno Diabetes Center Aarhus, Aarhus University Hospital, Arla Foods for Health (N/A), Augustinus Foundation (grant no. 19‐1349). JJH was supported by the NovoNordisk Foundation Center for Basic Metabolic Research.

## CONFLICT OF INTEREST

URM is employed at ARLA Foods Ingredients P/S and contributed to the study design and final draft of the manuscript, but was not involved in data collection, analysis or decision to publish. Furthermore, ARLA Foods did not have any influence on study conduct or interpretation. The remaining authors have no further conflict of interest to declare.

## ETHICS STATEMENT

Central Denmark Ethics Committee ID: 1‐10‐72‐69‐19.
